# Magnetoencephalography Atlas Viewer for Dipole Localization and Viewing

**DOI:** 10.3390/jimaging10040080

**Published:** 2024-03-28

**Authors:** N.C.d. Fonseca, Jason Bowerman, Pegah Askari, Amy L. Proskovec, Fabricio Stewan Feltrin, Daniel Veltkamp, Heather Early, Ben C. Wagner, Elizabeth M. Davenport, Joseph A. Maldjian

**Affiliations:** 1 MEG Center of Excellence, University of Texas Southwestern Medical Center, Dallas, TX 75390, USA; pegah.askari@utsouthwestern.edu (P.A.); amy.proskovec@utsouthwestern.edu (A.L.P.); fabricio.feltrin@utsouthwestern.edu (F.S.F.); daniel.veltkamp@utsouthwestern.edu (D.V.); heather.early@utsouthwestern.edu (H.E.); elizabeth.davenport@utsouthwestern.edu (E.M.D.); joseph.maldjian@utsouthwestern.edu (J.A.M.); 2 Advanced Neuroscience Imaging Research (ANSIR) Laboratory, University of Texas Southwestern Medical Center, Dallas, TX 75390, USA; jason.bowerman@utsouthwestern.edu (J.B.); ben.wagner@utsouthwestern.edu (B.C.W.); 3 Department of Radiology, University of Texas Southwestern Medical Center, Dallas, TX 75390, USA; 4 Biomedical Engineering Department, University of Texas Arlington, Arlington, TX 76019, USA; 5 Biomedical Engineering Department, University of Texas Southwestern Medical Center, Dallas, TX 75390, USA

**Keywords:** magnetoencephalography, dipole, labeling, automated, atlas, software application

## Abstract

Magnetoencephalography (MEG) is a noninvasive neuroimaging technique widely recognized for epilepsy and tumor mapping. MEG clinical reporting requires a multidisciplinary team, including expert input regarding each dipole’s anatomic localization. Here, we introduce a novel tool, the “Magnetoencephalography Atlas Viewer” (MAV), which streamlines this anatomical analysis. The MAV normalizes the patient’s Magnetic Resonance Imaging (MRI) to the Montreal Neurological Institute (MNI) space, reverse-normalizes MNI atlases to the native MRI, identifies MEG dipole files, and matches dipoles’ coordinates to their spatial location in atlas files. It offers a user-friendly and interactive graphical user interface (GUI) for displaying individual dipoles, groups, coordinates, anatomical labels, and a tri-planar MRI view of the patient with dipole overlays. It evaluated over 273 dipoles obtained in clinical epilepsy subjects. Consensus-based ground truth was established by three neuroradiologists, with a minimum agreement threshold of two. The concordance between the ground truth and MAV labeling ranged from 79% to 84%, depending on the normalization method. Higher concordance rates were observed in subjects with minimal or no structural abnormalities on the MRI, ranging from 80% to 90%. The MAV provides a straightforward MEG dipole anatomic localization method, allowing a nonspecialist to prepopulate a report, thereby facilitating and reducing the time of clinical reporting.

## 1. Introduction

Magnetoencephalography (MEG) is a noninvasive, FDA-approved functional neuroimaging technique that measures the magnetic field generated by the postsynaptic potential of pyramidal neurons, allowing for the study of neuronal activity with a high temporal resolution, typically exceeding the millisecond range, and a spatial resolution in the order from 2 to 3 mm [1,2,3]. The applications of MEG are vast, with rising demand for clinical as well as research purposes [4,5,6,7,8]. There is robust evidence demonstrating the advantages of MEG in clinical practice [9,10], with a well-established application in the epilepsy field, where it plays a key role in presurgical evaluation for identifying the potential epileptogenic zones and functional brain mapping of evoked responses that can include the estimation of the somatosensory, motor, auditory, visual, and language-evoked fields [3,6,7]. This results in less invasive surgery and improved clinical outcomes [9,10,11].

The clinical analysis of MEG data is based on a source-space approach [12]. Mathematical algorithms estimate the source of activity on Magnetic Resonance Imaging (MRI), combining neurophysiology with imaging studies, referred to as Magnetic Source Imaging (MSI) [1,13]. MSI relies on the accurate source localization of the captured signal on the patient’s anatomy, estimating the brain area generating the measured signal [12,13,14,15,16].

There are multiple distinct approaches available for MEG source localization. The most widely used and validated method for clinical purposes is equivalent current dipole (ECD) fitting [3,13]. The ECD model represents the location and direction of current flow. The model assumes that the data are coming from a single spot on the cortex [13], and it relies on the projection of a single-point dipole, meeting minimal predetermined statistical criteria, onto the patient’s MRI [13,17].

The general ECD analysis includes the visual detection of the interictal spikes and then the estimation of the ECD at a time point within the rise of the spike wave [17,18]. The dipole is displayed on the patient’s MRI, typically a T1 sequence, to represent the point of maximal activity at the chosen time point. A similar process is repeated for the localization of the eloquent cortex based on the averaged response [6,7]. Dipole clusters are groups of closely localized individual dipoles, which can provide valuable insights into underlying pathology [17]. Radiologic correlation is crucial in interpreting dipole clusters, and the presence of a contributory lesion in proximity to an MEG cluster can support its epileptogenicity [17,19]. Finally, the clinical report describes the brain structures where the dipoles are localized, along with their spatial relationship to lesions or eloquent areas [20].

While many papers have focused on automated pipelines for MEG source localization analysis [21,22,23,24], few have addressed automated anatomic identifications, despite the successful implementation in other modalities [25,26]. Maldjian et al. (2003) [25] demonstrated a successful automated method for performing hypothesis-driven inference from fMRI data using atlas-based masking. Taylor et al. (2021) [26] presented a semi-automated method of atlas-based anatomical labeling of stereoelectroencephalography electrode contacts. Nevertheless, we could not find a similar approach developed for the anatomical labeling of MEG dipoles.

In the process of assigning anatomical labels to regions depicted in T1-weighted MRIs, several specific procedures are requisite. The initial step involves brain extraction and segmentation. In brain extraction, the skull is eliminated from the image, thereby preserving solely the brain tissues and cerebrospinal fluid (CSF) [27]. In the segmentation, probability maps are generated to allocate to each voxel a likelihood of pertaining to distinct brain tissues, predominantly gray matter (GM), white matter (WM), and CSF [27].

The normalization of the MRI to standardized space plays a pivotal role in ensuring the consistency and accuracy of source reconstruction in neuroscientific research [28,29,30,31,32] and, consequently, in MEG dipole labeling. This critical procedure involves the transformation of an individual subject’s MRI to a standardized brain, facilitating comparisons and analyses across subjects with varying brain morphologies [31,32].

Numerous spatial normalization algorithms and brain templates have been developed for MRI studies focusing on neurologically healthy individuals. Commonly used spatial normalization methods are FLIRT (FMRIB’s Linear Image Registration Tool) [33,34,35] and FNIRT (FMRIB’s Nonlinear Image Registration Tool) [36] from the FMRIB (Functional Magnetic Resonance Imaging of the Brain) [36,37,38] Library and CAT12 (Computational Anatomy Toolbox, version 12) [39], an extension to SPM [40] 12 (Statistical Parametric Mapping 12).

Two commonly employed reference spaces for this purpose are the Talairach space [41] and the Montreal Neurological Institute (MNI) space [42]. The former is based on autopsy images of a single brain, while the latter relies on MRI scans from a substantial population of adult subjects.

Broadly, the MNI space delineates brain boundaries in millimeter coordinates from a designated origin point. However, it is essential to note that depending on the specific template utilized within the MNI space, a given set of coordinates may correspond to different anatomical structures [43].

The inception of the MNI305 Average Brain template marked a significant milestone in the field. This template was constructed from 305 T1-weighted MRI scans, thereby addressing the limitations inherent in using a single subject’s brain as the sole reference for brain mapping [42,44,45]. The contemporary standard in MNI templates is the International Consortium for Brain Mapping (ICBM) 152 [46,47], which represents the average of 152 normal MRI scans, carefully aligned to the MNI305 template using a nine-parameter affine transform. This template has been universally adopted as the standard reference by the International Consortium for Brain Mapping and has been consistently employed in neuroimaging research, starting with SPM99 onwards.

Templates lack anatomical labels. Consequently, atlases are commonly employed to delineate anatomically and functionally relevant regions of interest. The process typically involves normalizing structural MRI data to a standard atlas, such as MNI [42] atlases, and generating a transformation matrix. This matrix is subsequently employed to reverse-normalize the atlases to their original native MRI space.

In particular, the Talairach atlas [41,48,49] is renowned for providing detailed anatomical descriptions within the stereotaxic space, including delineations of Brodmann’s areas (BA) [50]. The Automated Anatomical Labeling (AAL) [51] atlas is also a valuable resource for anatomical localization within neuroimaging studies. The AAL atlas is based on anatomical parcellation of the MNI single-subject brain [51,52]. It was created with the purpose of labeling activations detected in PET or fMRI studies, aiming to clarify the relationship between coordinates and their anatomical labels. Also, it addresses the need for a consistent reference system in functional imaging research for reporting activation localization while recognizing that it does not account for inter-individual anatomical variability [32,43].

**Figure 1:**
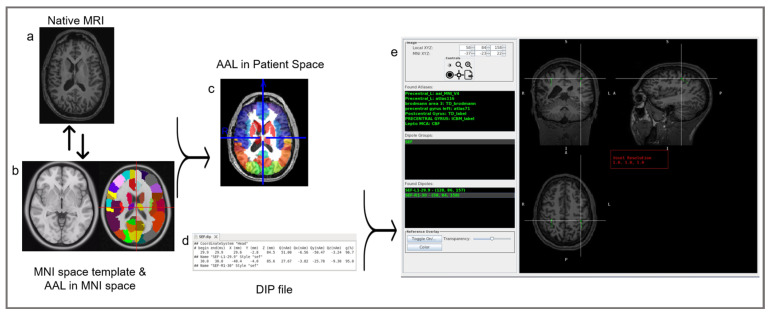
The MAV procedural workflow. On the left: MAV normalizes the patient’s native MRI (**a**) to the MNI space (**b**, **left**) and reverse-normalizes MNI atlases (**b**, **right**) to the native MRI (**c**). The color overlays in the template MRI (**b**, **right**) and the patient’s MRI (**c**) illustrate the parcellation of the MRI into regions of interest as determined by the selected atlas. In the middle: MAV utilizes the resulting patient’s MRI with overlaid MNI atlases (**c**) alongside the dipole file containing the dipoles’ coordinates (**d**) to match the spatial location with values from the NIfTI atlas files. Upon finding a matching value, it cross-references the corresponding labels within the lookup tables, thereby updating the displayed region list with the pertinent label names. On the right (**e**): a graphical user interface (GUI), detailed later, displaying individual dipoles (green), groups (green), coordinates, anatomical labels (green), and a tri-planar MRI view of the patient with dipole overlays (green square). In Found Atlases, the abbreviations are as follows: AAL, Automated Anatomical Labeling; MNI, Montreal Neurological Institute; TD, Talairach Atlas; ICBM, International Consortium for Brain Mapping; lepto, leptomeningeal; MCA, middle cerebral artery; CBF, cerebral blood flow. In dipole groups, the abbreviations are as follows: SEF: somatosensory-evoked field; L, left.

This paper presents a novel software application, the MEG Atlas Viewer (MAV), which normalizes the patients’ MRI, reverse-normalizes a variety of labeled atlases, applies the MEG-MRI coregistration transformation, and presents a list of identified MEG dipoles along with the corresponding brain region from a wide variety of atlases (Figure 1). The primary goal is to provide a semi-automated and accurate solution to facilitate the MEG dipole anatomic localization during MEG reporting.

## 2. Materials and Methods

The following subsections introduce the MAV program, its usage, and validation methods.

### 2.1. The MEG Atlas Viewer

To generate an automated atlas lookup for MEG dipoles, we developed the MAV. It presents a list of identified dipoles along with an assortment of atlases to choose from and displays the identified brain region of each MEG dipole. The MAV provides a scrollable tri-planar display of dipoles overlaid on the brain. The user can select a dipole, generated previously as part of the clinical workflow, and the MAV returns a list of atlas labels corresponding to that region. The MAV also provides an outline of the registered skull that can be toggled on and off to verify the quality of the normalization. The MAV was designed to read the MEGIN (Espoo, Finland) file structure. However, viewing the results can be performed independently of the manufacturer-provided software. The user selects the subject folder, which contains the MRI and the dipole locations in ‘.dip’ files (Supplementary Figure S1), a dipole data file type commonly generated by MEG systems. The dipole coordinates and dipole moments (Supplementary Figure S2) are used to spatially correlate its position with entries within the Neuroimaging Informatics Technology Initiative (NifTI) atlas file. Once a folder has been selected, the MAV automatically identifies the dipole files named specifically for each task (i.e., SEF.dip for somatosensory), the structural MRI, and the MEG-MRI coregistration matrix. For the MRI, the MAV input is Digital Imaging and Communications in Medicine (DICOM) images, which is the standard for medical imaging data interchange, and it automatically runs a dcm2nii to generate a NIfTI file, which is the format required for the normalization/registration routines.

### 2.2. Atlases

Atlas regions of interest (ROIs) are stored as unique integer values for each label in a NIfTI image. A corresponding text file lookup table contains the atlas label name and assigned integer value for that atlas. For example, utilizing the Montreal Neurological Institute (MNI) AAL [51] atlas, version 4, the voxel number 85 will return the following response:

85 “Temporal_Mid_L”.

**Figure 2:**
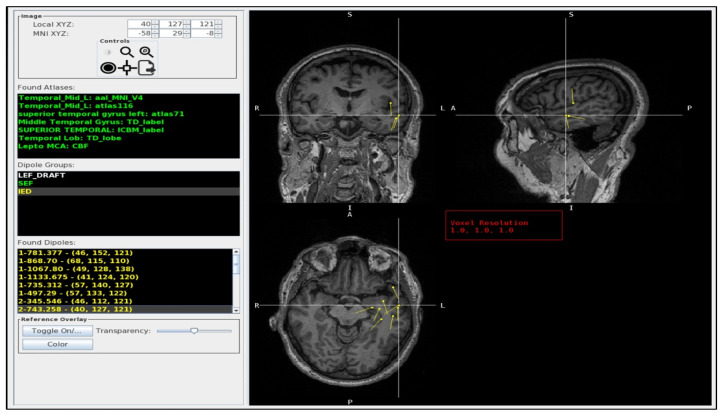
The MAV GUI. The left column lists the dipole crosshair coordinates, the atlas labels (Found Atlases), the available dipole groups (dipole groups), and the individual dipoles in that group (Found Dipoles). The Found Atlases section the different brain atlases and the corresponding labels (green) for the crosshair dipole. The Dipole group lists the .dip files found in the patients’ folder: LEF_DRAFT (white), SEF (green), IED (yellow). The Found Dipoles list the dipoles (yellow) found inside the selected folder (in the picture, the IED). The right side provides an interactive tri-planar MRI display (coronal: top left, sagittal: top right, and axial: bottom left) with color-coded dipole groups (yellow) and the ability to interrogate individual dipoles. In this example, the crosshair is centered on a dipole from the left temporal pole (superior temporal gyrus) in which there was full agreement between radiologists and the MAV. In Found Atlases, the abbreviation is as follows: Temporal_Mid_L, left middle temporal gyrus. In dipole groups, the abbreviations are as follows: LEF, language-evoked field; SEF: somatosensory-evoked field; IED, interictal epileptiform discharge; L, left. In the MRI slices, the abbreviations are as follows: R, right; L, left; S, superior; I, inferior; A, anterior; P, posterior.

This indicates that all voxels with a value of 85 represent the left middle temporal gyrus (Figure 2). The MAV was adapted from the Pickatlas [25] and contains 22 atlases (Supplementary Table S1) [48,49,51,53,54,55,56]. These include the MNI_AAL [51], version 4 of the “AAL_MNI_V4”, the same as the AAL [51] of 116 segmented structures “atlas116” from the Neurofunctional Imaging Group, the Individual Brain Atlases Using Statistical Mapping Software (IBASPM) [57] of 71 segmented structures “atlas71”, the ICBM [46] label “ICBM label” from the UCLA Brain Mapping Center, and the “Talairach Daemon (TD) lobe” from the Talairach Daemon [41,48,49].

As previously stated, the MAV uses the dipole values to match the spatial location with a value in the NIfTI atlas files. If the value has a corresponding label in the lookup tables, the displayed region list is updated with the appropriate label name. The displayed dipoles and brain regions are color-coded to simplify the distinction between different dipole groups and regions while being displayed concurrently. The atlas overlay transparency can be adjusted relative to the background MRI; the atlas presentation order can be customized based on user preference.

### 2.3. Normalization

The MAV implements three different normalization approaches, including FLIRT (FMRIB’s Linear Image Registration Tool) [33,34,35] and FNIRT (FMRIB’s nonlinear image registration tool) [36] methods from the FMRIB (Functional Magnetic Resonance Imaging of the Brain) Software Library [36,37,38], FSL version 5.0.11 [38] (https://fsl.fmrib.ox.ac.uk/fsl/fslwiki/, accessed 15 January 2024), and CAT12 (version 12 of the Computational Anatomy Toolbox) [39] (http://www.neuro.uni-jena.de/cat/, accessed 15 January 2024).

SPM and FSL are two widely used neuroimaging platforms that vary in accuracy depending on the tissue being evaluated [58]. Despite these differences, both platforms generally produce robust results. Our service defaults to using FLIRT due to its relatively fast processing time and satisfactory results, which make it suitable for routine analyses. However, users have the choice of selecting alternative normalization methods that meet their specific needs or preferences. FSL has been shown to be susceptible to image noise and nonhomogeneity in image intensity [59]. In such cases, SPM may be a more appropriate alternative.

The structural MRI is normalized to the MNI [42] atlas based on the selected method, generating a transformation matrix. This transformation matrix is then used to reverse-normalize the atlases to the native MRI space. The resulting normalized image and transformation matrix are saved to the subject directory for use with the dipole files.

### 2.4. Atlas Normalization Validation

**Figure 3:**
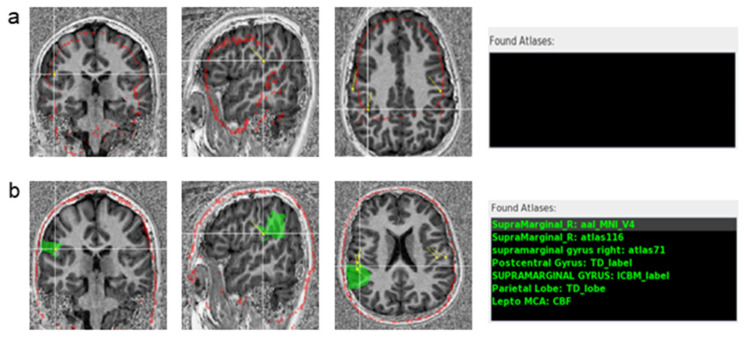
Skull registration validation on an MP2RAGE T1-weighted MRI. (**a**) Registration using FLIRT: the skull model (red outline) does not match the patient’s MRI. (**b**) Registration in the same subject using CAT12: the skull model (red outline) matches the skull on the patient’s MRI. The atlases’ labels (in green) for the selected dipole are displayed on the right of the image (Found Atlases). On the top, using FLIRT registration, which failed in this patient, no atlas was found. On the bottom, the Found Atlases section lists the different brain atlases and the corresponding labels (green) for the crosshair dipole, using CAT12. The selected dipole is correctly labeled by the AAL atlas as the right supramarginal gyrus, highlighted by the green overlay. In Found Atlases, the abbreviation is as follows: R, right.

The MAV provides the user with a skull overlay to quickly determine the quality of the normalization process (Figure 3). The skull outline is constructed based on the SPM MNI T1 atlas template that is reverse-normalized to the space of the structural T1. If the skull overlay does not align with the subject’s skull (Figure 3a), the quality of the atlas overlays may also be inadequate. The quality of atlas normalization can vary based on the T1-weighted image quality and normalization method. If the atlas overlays do not align, the user has the opportunity to select an alternative normalization method to improve the results (Figure 3b).

### 2.5. MEG Dipoles

Interictal discharges and evoked responses are modeled according to statistical criteria using the clinical MEGIN software, generating dipoles that best reflect the location of the activation. The files containing the list of dipoles are saved using a standardized naming convention inside the patient’s folder accessed by the MAV.

The MEGIN MEG-MRI coregistration affine matrices are stored in MEG Functional Image Files (FIF). The MAV automatically identifies and reads these files to compute the transformation matrix from the dipole coordinates to the voxel space of the T1-weighted image. The MAV graphical user interface (GUI) provides the user with the individual dipole label, coordinates, and tail vector. Each dipole is a 3D representation of the generating source in the brain. Multiple lists of dipoles may exist depending on the functional tasks performed. The dipoles are color-coded to distinguish them by their respective tasks. During development, the dipoles and their tails were validated against the MEGIN clinical MRiLab viewer from the Neuromag Data Analysis Software, version 3.4.4 (DANA, MEGIN OY) to ensure appropriate placement.

The viewer can switch between neurological (right on right) and radiological (right on left) orientations. The native T1-weighted image is displayed with the selected reverse-normalized atlases and dipoles, which have been converted to the native MRI space.

### 2.6. Testing the Performance of the Viewer

To validate the atlas, we included ten patients (273 dipoles) with both MEG scans and a T1-weighted MRI performed as part of their standard clinical care.

To establish an abnormal epileptic activity model, a source model must pass two tests: (1) an initial error test that includes chi-square and goodness of fit (GOF) evaluation, and (2) a subsequent significance test, including confidence volume (CV), or volume of error, of the dipole localization, as well as a signal-to-noise ratio (SNR) [17]. In this study, all of the dipoles included were previously accepted as sECD models that met these criteria: reduced chi-square < 2, GOF ≥ 80%, CV < 1000 mm^3^, and dipole strength between 100 and 500 nAm as proposed in the literature [17].

We used two normalization methods, FLIRT and CAT12, and we compared the radiologists’ analysis to the AAL MNI V4 atlas labeling. We validated the atlas’ performance against ground truth, which was established by a consensus of three board-certified radiologists, with fellowship training in neuroradiology and having accrued a minimum of two years of experience in MEG dipole analysis. Prior to the evaluation, a clear threshold was set for achieving consensus on the classification of each dipole. This threshold was defined as a minimum of 66% agreement among the neuroradiologists, which translates to at least two of the three experts concurring on each evaluation.

The neuroradiologists evaluated the atlas’ performance by independently identifying each dipole’s location and reporting when the dipole was in WM or CSF. In cases where the dipole localized in a sulcus, they evaluated whether they would consider one of the adjacent gyri.

## 3. Results

A representative image of the GUI is shown in Figure 2. It displays a list of the dipole groups, e.g., interictal epileptiform discharges (IED), the language-evoked field (LEF), and the somatosensory-evoked field (SEF), and a list of the individual dipoles in that group, including the user-defined dipole label and voxel coordinates. By choosing a dipole from the list, the dipole is centered in the MRI image view and highlighted by the crosshair. The GUI lists the dipole crosshair coordinates and the atlas labels with the corresponding atlas name.

The GUI also provides some control buttons. The ‘Toggle On’ button displays the MNI skull ROI overlaid on the subject MRI for atlas validation (Figure 3). Selecting a label from a specific atlas makes it also possible to visualize the atlas overlay on the MRI (Figure 3b).

### 3.1. Usage of the Program Tools

We ran the program in 273 dipoles from 10 patients (5 males and 5 females; mean age 28.9 y, ±18.0 y) using FLIRT and CAT12 normalization methods. T1-weighted MPRAGE [60,61] and Magnetization-Prepared 2 Rapid Gradient-Echo (MP2RAGE) [62,63] sequences were used for normalization. Seven patients had MRIs with non-evident or minimal signal changes, without surgical or structural abnormalities. Three patients had structural changes, namely, resection cavities or encephalomalacia. These alterations encompassed (a) left cystic encephalomalacia with postsurgical changes stemming from a previous arachnoid cyst resection, (b) cystic encephalomalacia affecting the right frontal and temporal lobes as sequelae of prior head trauma, and (c) postsurgical changes originating from a right anterior temporal lobectomy with two additional surgical interventions, broadening the resection margins and encompassing the inferior temporal gyrus and posterior insula.

FSL FLIRT registration failed in three patients (71 dipoles) with the MP2RAGE sequences (Figure 3a), in which the skull mask had poor registration to the patients’ native MRI. However, in these cases, CAT12 registration was successful (Figure 3b).

FLIRT normalization, however, ran faster (20–120 s) than CAT12 (10–35 min). The number of dipoles being analyzed interfered with time variations within the methods.

### 3.2. Performance of the Program

To test the performance of the program, we compared the AAL atlas’ labeling of the 273 dipoles against the radiologists’ consensus. A total of 71 dipoles were excluded from the FLIRT analysis due to the failure of the package during the segmentation and normalization method. A total of 33 dipoles were unlabeled (i.e., no AAL atlas label was found at the coordinates) by FLIRT and 31 by CAT12 (Table 1). The unlabeled dipoles were due to their localization in anatomical structures not being covered by the AAL atlas, including regions such as WM and CSF. In most of these cases, the ICBM_label atlas correctly labeled the dipole (Supplementary Figure S3). Notably, although some dipoles were situated within WM or CSF, they were in proximity to GM structures, leading to their inclusion in the analysis. Additionally, we excluded two dipoles positioned centrally within WM or CSF, in a location where it was difficult to identify the corresponding region.

Considering all of the dipoles labeled by FLIRT (150) and CAT12 (241), there was a concordance between the radiologists’ ground truth and MAV labeling in approximately 83% (125/150) and 78% (187/224) of the cases, respectively. The concordance with the AAL atlas was higher where there were minimal or no structural abnormalities on the MRI, reaching 90% (65/72) for FLIRT and 80% (129/161) for CAT12 (Table 1).

Dipoles located within or bordering a sulcus, in WM, or CSF were more susceptible to being unlabeled by the AAL atlas or exhibiting discordance with the radiology consensus, particularly in MRIs with significant structural abnormalities. When dipoles were located within a sulcus, the radiologist accepted one of the adjacent gyri as the correct labeling. Additionally, these cases were especially susceptible to variations in the labeling depending on the segmentation method.

AAL atlas labeling discrepancies when using FLIRT or CAT12 occurred in 18.5% of the non-excluded dipoles (270). In 86% of these cases, the dipole was in or bordering a sulcus, in WM, or CSF. In 60% of those, only one method labeled the dipole. For the remaining 40%, the radiologists’ consensus was most concordant with AAL labeling using FLIRT (60%). It is essential to point out that in some of these cases, other atlases were concordant with the radiologist’s consensus when using CAT12, such as the ICBM atlas. CAT12 analysis included three more patients with minimal or no structural abnormalities, in which FLIRT/FSL normalization failed; nevertheless, excluding these patients, the concordance with CAT12 did not vary significantly, remaining at approximately 81%. CAT12 also identified a higher percentage of dipoles than FLIRT.

Variation between normalization methods and the labeling was also observed when the dipoles were over the atlas parcellations’ boundaries. However, most differences had no clinical significance since both accounted for the same region referred to in the clinical context.

Additionally, at least three patients had dipoles in subcortical structures, including the hippocampus, fusiform gyrus, and para-hippocampus, which were located with satisfactory accuracy and thus were not deemed as a limitation factor.

## 4. Discussion

Clinical MEG is a functional neuroimaging technique that demands high expertise and labor. Typically, the most time-consuming aspect of clinical reporting involves the interpretation of the neurophysiologic data and the selection of epileptogenic activity. While anatomical structural analysis is generally considered a relatively straightforward task, particularly for radiologists, MEG reports frequently involve summarizing location information, sometimes of numerous dipoles, and relating them to other technical and clinical aspects. The primary goal of the MAV is to enhance the efficiency and automation of the dipole anatomical identification analysis in MEG, allowing a nonspecialist to accurately integrate all of the information and pregenerate a report, thereby reducing the time spent by the interpreting physician during MEG reporting.

**Figure 4:**
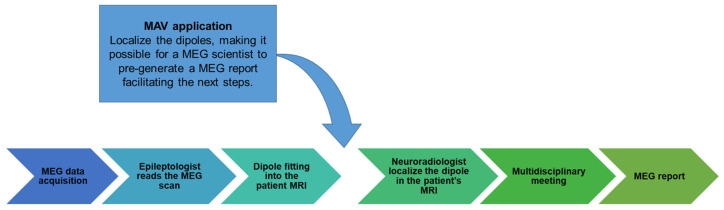
Clinical workflow. This flow diagram illustrates the clinical steps involved in obtaining the MEG report at our institution and the integration of the MAV into this clinical workflow. The arrow in the figure indicates the process by which the MAV application is utilized. The MAV application’s role is to localize the dipoles, making it easier for a scientist to pre-generate an MEG report to facilitate subsequent steps in the patient’s diagnosis and treatment planning.

At our center, a multidisciplinary virtual meeting approach is employed to review the clinical MEG studies. Participants include MEG scientists, neurophysiologists, and neuroradiologists. The MAV permits MEG scientists and technologists to prepopulate the report with suggested regions, allowing for streamlined reviewing and reporting by the neuroradiologists (Figure 4). The MEG scientists share the MEGIN clinical view using the MEGIN software, while other study members can concurrently and independently review and interrogate the findings using the MAV. It also facilitates remote viewing of the results independent of the current manufacturer-provided software.

In the MEGIN software system, analyzing patients’ dipoles within their MRI scans involves several steps. Firstly, users must initiate the MEG-MRI integration application. Subsequently, they must navigate the patient’s folder to locate and open the MRI file. Following this, users must access the dipole list viewer (object tree) and manually search for each dipole file that needs to be visualized, subsequently importing them.

In contrast, the process is notably more streamlined within the MAV. Users simply need to search for the patient folder and click on it. The viewer then autonomously locates and opens both the patient’s MRI scan and all associated dipole group files. This approach significantly enhances user-friendliness and expedites the analysis process. This is preferable to viewing the DICOMs generated through the clinical software and exported to the clinical PACs, as these typically have the dipoles “burned in” which severely limits any interactivity on the PACs.

Regarding the quality of the normalization, in most cases, FLIRT was successful. However, in some cases of MP2RAGE images, FLIRT normalization failed. MP2RAGE, a modified version of MPRAGE sequences, is an inversion recovery sequence that acquires two rapid gradient echoes at two inversion times [62,63,64]. The aim of MP2RAGE is to enhance the T1w contrast by removing the proton density component, but this also amplifies the noise [62,63]. The resulting image has an improved T1 signal but with increased noise in the background and cavities of the skull, making the delineation of structures difficult and problematic for some registration and segmentation algorithms [65].

Segmentation by FSL is based on image intensities [66], whereas SPM12/CAT12 uses a template image as a reference for brain tissue. The MAV was designed to enable the user to choose from different normalization packages. In general, the alternative CAT12 normalization effectively solved the normalization issues without requiring any further processing. To avoid issues regarding the segmentation/brain extraction step when launching the MAV, we recommend avoiding FSL-based segmentation methods when the available T1-weighted image is an MP2RAGE sequence.

In order to validate the MAV, a consensus of three neuroradiologists was used to establish a ground truth of the labeling. The concordance between their labeling and the AAL atlas was high, especially when analyzing MRIs with minimal or no structural abnormalities. Variations in the MAV labeling associated with different normalization methods mainly occurred when the dipoles were situated in boundary areas. In these situations, the dipoles were also more susceptible to variations between atlases using the same normalization method, for example, being unlabeled by the AAL atlas or discordant with the radiology consensus.

While a formal analysis of other atlases was not conducted, it was nonetheless apparent that in instances involving boundary areas, the labeling within the same atlas exhibited variations, even when employing an identical normalization method. Interestingly, one of the alternative atlases in these cases frequently recognized the gyrus identified as the correct one by the consensus. This observation suggests that users may identify the most convenient atlas for their specific needs with continued use. It is also worth noting that in situations where dipoles are not labeled due to localization in WM (Supplementary Figure S3) or CSF but border cortical areas of interest, the user can drag the cursor to the nearest GM region to the dipole and verify the labeling outcome using atlases like AAL or ICBM label.

**Figure 5:**
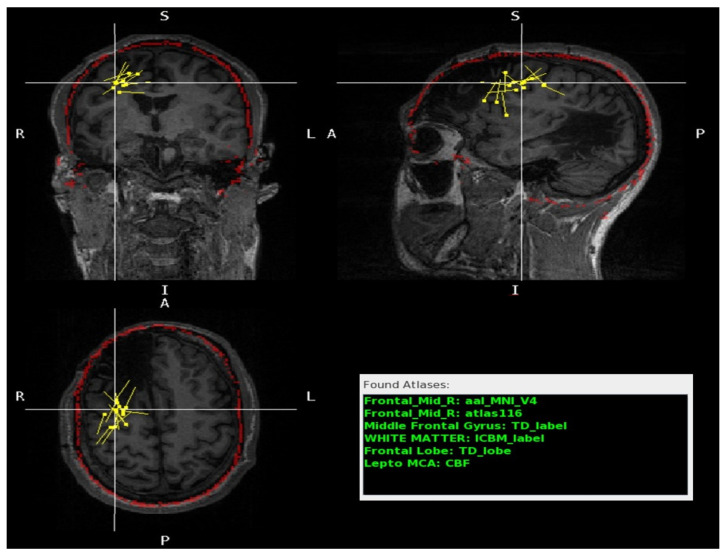
A patient with right frontal and temporal cystic encephalomalacia. The MRI scan is shown in three different planes: coronal (**top left**), sagittal (**top right**), and axial (**bottom left**). The red outline indicates the contour of the skull model overlay and demonstrates satisfactory patient anatomy registration. The yellow dots represent the dipoles modeled. The crosshair dipole is close to the cavity but in a preserved anatomic area. The Found Atlases section (**bottom right**) lists the different brain atlases and the corresponding labels (green) for the crosshair dipole. The atlas labels matched the radiologists’ labels with minimal discordance, predominantly in areas at the border of the cavity. The atlas labels matched the radiologists’ label, with minimal discordance, predominantly in areas at the border of the cavity. In Found Atlases, the abbreviation is as follows: Frontal Mid, middle frontal gyrus.

One of the limitations of the MAV is that some dipoles were mislabeled in the presence of significant structural changes. The accuracy varied depending on the size of the structural cavity and the degree of distortion from the normal anatomy (Figure 5). Standard templates, although widely used in the field and adequate for MRI with minimal structural abnormalities, may not accurately represent the unique anatomical variations present in individuals with more extensive structural abnormalities, such as those who had undergone surgical resections. For instance, the case with the worst concordance was a patient who underwent an extensive right temporal resection covering the right temporal pole. In this patient, the skull overlay was adequate. The atlas overlay was accurately superimposed, with the corresponding anatomical regions correctly aligned. However, it was inadequate for the patient’s specific anatomy, as it labeled areas that had been excised based on standard templates and inaccurately localized some of the dipoles. For example, the inferior portions of the insula, which are usually covered by the temporal pole, were exposed, resulting in dipoles modeled in that region being identified as the temporal gyrus.

In the remaining two cases where there were significant structural changes, the majority of the dipoles were situated in regions that exhibited minimal or no deviation from the standard template, e.g., in the proximity of the cavity (Figure 5). It is important to exercise caution when interpreting results from patients with significant structural abnormalities.

We acknowledge an additional limitation of our study. While we included adult and pediatric patients, our cohort comprised patients aged ten years and above. In our MEG service, the number of adult patients is significantly higher than that of pediatric patients. Although we have utilized the software for patients as young as three years old and have obtained satisfactory results, this younger cohort was not represented in our study sample. Additionally, we have no prior experience with neonatal MEG. Nevertheless, standard MRI template brain volumes specifically designed for pediatric data [67] could be seamlessly integrated into the software, offering a viable solution for MEG services catering to a substantial pediatric demographic. Future endeavors will prioritize the incorporation of pediatric MNI templates into our software and the subsequent assessment of their efficacy and reliability in accommodating younger patient populations.

## 5. Conclusions

In this study, we have demonstrated MAV, a software application that generates automated atlas labeling for MEG dipoles. The program provides an intuitive, user-friendly, and interactive viewer for dipole visualization. We tested the MAV across various clinically relevant scenarios and compared it to expert neuroradiologists’ analysis, demonstrating high concordance. The MAV provides an effective and efficient tool to localize and label MEG dipoles, thereby facilitating clinical MEG reporting.

## Figures and Tables

**Table 1:** Labeling accuracy for MAV.

	All MRIs		MRIs with Minimal or No Structural Abnormalities	
	Concordant Dipoles	Unlabeled Dipoles	Concordant Dipoles	Unlabeled Dipoles
FLIRT	83%	18%	90%	11%
	(125/150)	(33/185)	(65/72)	(9/82)
CAT12	78%	11%	80%	5%
	(187/241)	(31/273)	(129/161)	(9/170)

## Data Availability

The data are not publicly available due to patient privacy; however, the deidentified data supporting this study’s findings are available from the corresponding author upon reasonable request and formal data use agreement. The MAV source code is available on our Git repository site at https://git.biohpc.swmed.edu/s190502/mav, accessed 22 January 2024.

## Supplementary Materials

The following supporting information can be downloaded at: https://www.mdpi.com/article/10.3390/jimaging10040080/s1, Figure S1. The screenshot illustrates the file structure within our MEG system; Figure S2. Screenshot of a .dip file generated by our MEG software, with the dipole’s x, y, and z coordinates circled in blue and the dipole’s moments circled in orange, which are the values MAV uses to match with the MRI; Figure S3. MPRAGE T1-weighted MRI with the crosshair centered on a dipole localized within the white matter (WM). Table S1. List of all available atlases in MAV and their labeling systems.

## Abbreviations

The following abbreviations are used in this manuscript:

AAL	Automated Anatomical Labeling
CAT12	version 12 of the Computational Anatomy Toolbox
FLIRT	FMRIB’s Linear Image Registration Tool
MEG	Magnetoencephalography

## References

[1] Hämäläinen M., Hari R., Ilmoniemi R.J., Knuutila J., Lounasmaa O.V. (1993). Magnetoencephalography—Theory, instrumentation, and applications to noninvasive studies of the working human brain. Rev. Mod. Phys..

[2] Murakami S., Okada Y. (2006). Contributions of principal neocortical neurons to magnetoencephalography and electroencephalography signals. J. Physiol..

[3] Hari R., Baillet S., Barnes G., Burgess R., Forss N., Gross J., Hämäläinen M., Jensen O., Kakigi R., Mauguière F. (2018). IFCN-endorsed practical guidelines for clinical magnetoencephalography (MEG). Clin. Neurophysiol..

[4] Stufflebeam S.M., Tanaka N., Ahlfors S.P. (2009). Clinical applications of magnetoencephalography. Hum. Brain Mapp..

[5] Kim J.A., Davis K.D. (2021). Magnetoencephalography: Physics, techniques, and applications in the basic and clinical neurosciences. J. Neurophysiol..

[6] Burgess R.C., Funke M.E., Bowyer S.M., Lewine J.D., Kirsch H.E., Bagić A.I., ACMEGS Clinical Practice Guideline (CPG) Committee (2011). American Clinical Magnetoencephalography Society Clinical Practice Guideline 2: Presurgical functional brain mapping using magnetic evoked fields. J. Clin. Neurophysiol..

[7] Bowyer S.M., Pang E.W., Huang M., Papanicolaou A.C., Lee R.R. (2020). Presurgical functional mapping with magnetoencephalography. Neuroimaging Clin..

[8] Fred A.L., Kumar S.N., Kumar Haridhas A., Ghosh S., Purushothaman Bhuvana H., Sim W.K.J., Vimalan V., Givo F.A.S., Jousmäki V., Padmanabhan P. (2022). A Brief Introduction to Magnetoencephalography (MEG) and Its Clinical Applications. Brain Sci..

[9] Murakami H., Wang Z.I., Marashly A., Krishnan B., Prayson R.A., Kakisaka Y., Mosher J.C., Bulacio J., Gonzalez-Martinez J.A., Bingaman W.E. (2016). Correlating magnetoencephalography to stereo-electroencephalography in patients undergoing epilepsy surgery. Brain.

[10] Vadera S., Jehi L., Burgess R.C., Shea K., Alexopoulos A.V., Mosher J., Gonzalez-Martinez J., Bingaman W. (2013). Correlation between magnetoencephalography-based “clusterectomy” and postoperative seizure freedom. Neurosurg. Focus.

[11] Mohamed I.S., Toffa D.H., Robert M., Cossette P., Bérubé A.-A., Saint-Hilaire J.-M., Bouthillier A., Nguyen D.K. (2020). Utility of magnetic source imaging in nonlesional focal epilepsy: A prospective study. Neurosurg. Focus.

[12] Hämäläinen M., Huang M., Bowyer S.M. (2020). Magnetoencephalography signal processing, forward modeling, inverse source imaging, and coherence analysis. Neuroimaging Clin..

[13] Ray A., Bowyer S.M. (2010). Clinical applications of magnetoencephalography in epilepsy. Ann. Indian Acad. Neurol..

[14] Burgess R.C. (2020). MEG for greater sensitivity and more precise localization in epilepsy. Neuroimaging Clin..

[15] Tenney J.R., Fujiwara H., Rose D.F. (2020). The value of source localization for clinical magnetoencephalography: Beyond the equivalent current dipole. J. Clin. Neurophysiol..

[16] Mosher J.C., Funke M.E. (2020). Towards Best Practices in Clinical Magnetoencephalography: Patient Preparation and Data Acquisition. J. Clin. Neurophysiol..

[17] Laohathai C., Ebersole J.S., Mosher J.C., Bagić A.I., Sumida A., Von Allmen G., Funke M.E. (2021). Practical Fundamentals of Clinical MEG Interpretation in Epilepsy. Front. Neurol..

[18] Bagic A.I., Knowlton R.C., Rose D.F., Ebersole J.S., ACMEGS Clinical Practice Guideline (CPG) Committee (2011). American clinical magnetoencephalography society clinical practice guideline 1: Recording and analysis of spontaneous cerebral activity. J. Clin. Neurophysiol..

[19] Widjaja E., Otsubo H., Raybaud C., Ochi A., Chan D., Rutka J.T., Snead O.C., Halliday W., Sakuta R., Galicia E. (2008). Characteristics of MEG and MRI between Taylor’s focal cortical dysplasia (type II) and other cortical dysplasia: Surgical outcome after complete resection of MEG spike source and MR lesion in pediatric cortical dysplasia. Epilepsy Res..

[20] Burgess R.C. (2020). MEG Reporting. J. Clin. Neurophysiol..

[21] Bock E., Baillet S. MEG-Clinic: A Comprehensive Software Solution for Routine MEG Analysis. Proceedings of the 17th International Conference on Biomagnetism Advances in Biomagnetism–Biomag2010.

[22] Capilla A., Arana L., García-Huéscar M., Melcón M., Gross J., Campo P. (2022). The natural frequencies of the resting human brain: An MEG-based atlas. NeuroImage.

[23] Tait L., Özkan A., Szul M.J., Zhang J. (2021). A systematic evaluation of source reconstruction of resting MEG of the human brain with a new high-resolution atlas: Performance, precision, and parcellation. Hum. Brain Mapp..

[24] Hirano R., Emura T., Nakata O., Nakashima T., Asai M., Kagitani-Shimono K., Kishima H., Hirata M. (2022). Fully-Automated Spike Detection and Dipole Analysis of Epileptic MEG Using Deep Learning. IEEE Trans. Med. Imaging.

[25] Maldjian J.A., Laurienti P.J., Kraft R.A., Burdette J.H. (2003). An automated method for neuroanatomic and cytoarchitectonic atlas-based interrogation of fMRI data sets. Neuroimage.

[26] Taylor K.N., Joshi A.A., Hirfanoglu T., Grinenko O., Liu P., Wang X., Gonzalez-Martinez J.A., Leahy R.M., Mosher J.C., Nair D.R. (2021). Validation of semi-automated anatomically labeled SEEG contacts in a brain atlas for mapping connectivity in focal epilepsy. Epilepsia Open.

[27] Antonopoulos G., More S., Raimondo F., Eickhoff S.B., Hoffstaedter F., Patil K.R. (2023). A systematic comparison of VBM pipelines and their application to age prediction. Neuroimage.

[28] Valdés-Hernández P.A., von Ellenrieder N., Ojeda-Gonzalez A., Kochen S., Alemán-Gómez Y., Muravchik C., Valdés-Sosa P.A. (2009). Approximate average head models for EEG source imaging. J. Neurosci. Methods.

[29] Vinding M.C., Oostenveld R. (2022). Sharing individualised template MRI data for MEG source reconstruction: A solution for open data while keeping subject confidentiality. NeuroImage.

[30] Klein A., Andersson J., Ardekani B.A., Ashburner J., Avants B., Chiang M.-C., Christensen G.E., Collins D.L., Gee J., Hellier P. (2009). Evaluation of 14 nonlinear deformation algorithms applied to human brain MRI registration. NeuroImage.

[31] Ashburner J., Friston K.J. (1999). Nonlinear spatial normalization using basis functions. Hum. Brain Mapp..

[32] Crinion J., Ashburner J., Leff A., Brett M., Price C., Friston K. (2007). Spatial normalization of lesioned brains: Performance evaluation and impact on fMRI analyses. NeuroImage.

[33] Jenkinson M., Bannister P., Brady M., Smith S. (2002). Improved Optimization for the Robust and Accurate Linear Registration and Motion Correction of Brain Images. NeuroImage.

[34] Jenkinson M., Smith S. (2001). A global optimisation method for robust affine registration of brain images. Med. Image Anal..

[35] Greve D.N., Fischl B. (2009). Accurate and robust brain image alignment using boundary-based registration. NeuroImage.

[36] Woolrich M.W., Jbabdi S., Patenaude B., Chappell M., Makni S., Behrens T., Beckmann C., Jenkinson M., Smith S.M. (2009). Bayesian analysis of neuroimaging data in FSL. Neuroimage.

[37] Smith S.M., Jenkinson M., Woolrich M.W., Beckmann C.F., Behrens T.E., Johansen-Berg H., Bannister P.R., De Luca M., Drobnjak I., Flitney D.E. (2004). Advances in functional and structural MR image analysis and implementation as FSL. Neuroimage.

[38] Jenkinson M., Beckmann C.F., Behrens T.E.J., Woolrich M.W., Smith S.M. (2012). FSL. NeuroImage.

[39] Gaser C., Dahnke R., Thompson P.M., Kurth F., Luders E., Alzheimer’s Disease Neuroimaging Initiative (2022). CAT—A Computational Anatomy Toolbox for the Analysis of Structural MRI Data. Neuroscience.

[40] Friston K.J. (1994). Statistical parametric mapping. Functional Neuroimaging: Technical Foundations.

[41] Talairach J., Tournoux P., Rayport M. (1988). Co-Planar Stereotaxic Atlas of the Human Brain: 3-Dimensional Proportional System: An Approach to Cerebral Imaging.

[42] Evans A.C., Collins D.L., Mills S.R., Brown E.D., Kelly R.L., Peters T.M. 3D statistical neuroanatomical models from 305 MRI volumes. Proceedings of the 1993 IEEE Conference Record Nuclear Science Symposium and Medical Imaging Conference.

[43] Brett M., Johnsrude I.S., Owen A.M. (2002). The problem of functional localization in the human brain. Nat. Rev. Neurosci..

[44] Evans A.C., Marrett S., Neelin P., Collins L., Worsley K., Dai W., Milot S., Meyer E., Bub D. (1992). Anatomical mapping of functional activation in stereotactic coordinate space. NeuroImage.

[45] Chau W., McIntosh A.R. (2005). The Talairach coordinate of a point in the MNI space: How to interpret it. NeuroImage.

[46] Mazziotta J., Toga A., Evans A., Fox P., Lancaster J., Zilles K., Woods R., Paus T., Simpson G., Pike B. (2001). A probabilistic atlas and reference system for the human brain: International Consortium for Brain Mapping (ICBM). Philos. Trans. R. Soc. London. Ser. B Biol. Sci..

[47] Mazziotta J.C., Toga A.W., Evans A., Fox P., Lancaster J. (1995). A Probabilistic Atlas of the Human Brain: Theory and Rationale for Its Development. NeuroImage.

[48] Lancaster J.L., Woldorff M.G., Parsons L.M., Liotti M., Freitas C.S., Rainey L., Kochunov P.V., Nickerson D., Mikiten S.A., Fox P.T. (2000). Automated Talairach Atlas labels for functional brain mapping. Hum. Brain Mapp..

[49] Lancaster J.L., Rainey L.H., Summerlin J.L., Freitas C.S., Fox P.T., Evans A.C., Toga A.W., Mazziotta J.C. (1997). Automated labeling of the human brain: A preliminary report on the development and evaluation of a forward-transform method. Hum. Brain Mapp..

[50] Judaš M., Cepanec M., Sedmak G. (2012). Brodmann’s map of the human cerebral cortex—Or Brodmann’s maps?. Transl. Neurosci..

[51] Tzourio-Mazoyer N., Landeau B., Papathanassiou D., Crivello F., Etard O., Delcroix N., Mazoyer B., Joliot M. (2002). Automated Anatomical Labeling of Activations in SPM Using a Macroscopic Anatomical Parcellation of the MNI MRI Single-Subject Brain. NeuroImage.

[52] Tzourio N., Petit L., Mellet E., Orssaud C., Crivello F., Benali K., Salamon G., Mazoyer B. (1997). Use of anatomical parcellation to catalog and study structure-function relationships in the human brain. Hum. Brain Mapp..

[53] Oishi K., Zilles K., Amunts K., Faria A., Jiang H., Li X., Akhter K., Hua K., Woods R., Toga A.W. (2008). Human brain white matter atlas: Identification and assignment of common anatomical structures in superficial white matter. Neuroimage.

[54] Figley T.D., Mortazavi Moghadam B., Bhullar N., Kornelsen J., Courtney S.M., Figley C.R. (2017). Probabilistic White Matter Atlases of Human Auditory, Basal Ganglia, Language, Precuneus, Sensorimotor, Visual and Visuospatial Networks. Front. Hum. Neurosci..

[55] Figley T.D., Bhullar N., Courtney S.M., Figley C.R. (2015). Probabilistic atlases of default mode, executive control and salience network white matter tracts: An fMRI-guided diffusion tensor imaging and tractography study. Front. Hum. Neurosci..

[56] Dunas T., Wahlin A., Ambarki K., Zarrinkoob L., Malm J., Eklund A. (2017). A Stereotactic Probabilistic Atlas for the Major Cerebral Arteries. Neuroinformatics.

[57] Alemán-Gómez Y. IBASPM: Toolbox for automatic parcellation of brain structures. Proceedings of the 12th Annual Meeting of the Organization for Human Brain Mapping.

[58] Tudorascu D.L., Karim H.T., Maronge J.M., Alhilali L., Fakhran S., Aizenstein H.J., Muschelli J., Crainiceanu C.M. (2016). Reproducibility and Bias in Healthy Brain Segmentation: Comparison of Two Popular Neuroimaging Platforms. Front. Neurosci..

[59] Kazemi K., Noorizadeh N. (2014). Quantitative Comparison of SPM, FSL, and Brainsuite for Brain MR Image Segmentation. J. Biomed. Phys. Eng..

[60] Mugler III J.P., Brookeman J.R. (1990). Three-dimensional magnetization-prepared rapid gradient-echo imaging (3D MP RAGE). Magn. Reson. Med..

[61] Mugler III J.P., Brookeman J.R. (1991). Rapid three-dimensional T1-weighted MR imaging with the MP-RAGE sequence. J. Magn. Reson. Imaging.

[62] Van de Moortele P.-F., Auerbach E.J., Olman C., Yacoub E., Uğurbil K., Moeller S. (2009). T1 weighted brain images at 7 Tesla unbiased for Proton Density, T2⁎ contrast and RF coil receive B1 sensitivity with simultaneous vessel visualization. Neuroimage.

[63] Marques J.P., Kober T., Krueger G., van der Zwaag W., Van de Moortele P.-F., Gruetter R. (2010). MP2RAGE, a self bias-field corrected sequence for improved segmentation and T1-mapping at high field. Neuroimage.

[64] Marques J.P., Gruetter R. (2013). New developments and applications of the MP2RAGE sequence-focusing the contrast and high spatial resolution R1 mapping. PLoS ONE.

[65] O’Brien K.R., Kober T., Hagmann P., Maeder P., Marques J., Lazeyras F., Krueger G., Roche A. (2014). Robust T1-weighted structural brain imaging and morphometry at 7T using MP2RAGE. PLoS ONE.

[66] Choi U.S., Kawaguchi H., Matsuoka Y., Kober T., Kida I. (2019). Brain tissue segmentation based on MP2RAGE multi-contrast images in 7 T MRI. PLoS ONE.

[67] Fonov V., Evans A.C., Botteron K., Almli C.R., McKinstry R.C., Collins D.L., Brain Development Cooperative G. (2011). Unbiased average age-appropriate atlases for pediatric studies. Neuroimage.

