# Supplementary material for: Magnetoencephalography Atlas Viewer for Dipole Localization and Viewing
Source: J Imaging. 2024 Mar 28;10(4):80. doi: 10.3390/jimaging10040080 (PMC11051542; doi:10.3390/jimaging10040080)
Supplement: Supplementary file 1 [file jimaging-10-00080-s001.zip › jimaging-2874943-supplementary.pdf]

Supplementary Material

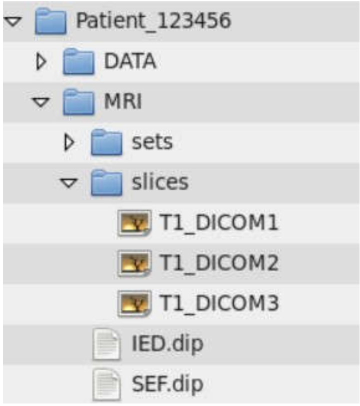

Supplementary Figure 1. The screenshot illustrates the file structure within our MEG system. Specifically, the dipole files are in the .dip format within the MRI folder, and the subfolder titled "slices" houses the DICOM files utilized by MAV. IED, Interictal Epileptiform Discharge; SEF, Somatosensory Evoked Field.

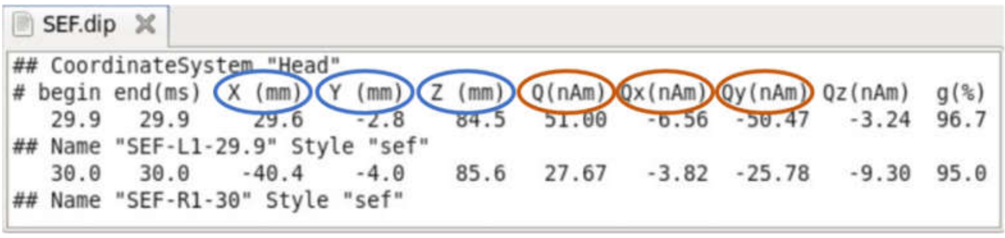

```
SEF.dip
## CoordinateSystem "Head"
# begin end(ms)  X (mm)  Y (mm)  Z (mm)  Q(nAm)  Qx(nAm)  Qy(nAm)  Qz(nAm)  g(%)
   29.9   29.9    29.6    -2.8    84.5    51.00   -6.56   -50.47    -3.24   96.7
## Name "SEF-L1-29.9" Style "sef"
   30.0   30.0   -40.4    -4.0    85.6    27.67   -3.82   -25.78    -9.30   95.0
## Name "SEF-R1-30" Style "sef"
```

Supplementary Figure 2. Screenshot of a .dip file generated by our MEG software, with the dipole's x, y, and z coordinates circled in blue and the dipole's moments circled in orange, which are the values MAV uses to match with the MRI. SEF, somatosensory evoked field; L, left; R right; mm, millimeters; Q, charge; nAm, Nano amperes; g, g-value.

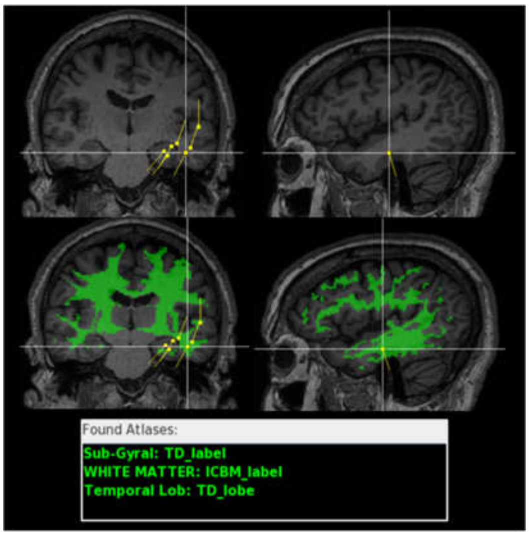

Supplementary Figure 3. MPRAGE T1-weighted MRI with the crosshair centered on a dipole localized within the white matter (WM). The MRI was segmented using the FLIRT method. The scan is shown in coronal (left) and sagittal (right) views. The top of the image shows only the dipoles, while the bottom shows the ICBM label WM atlas parcellation overlays the dipole. The AAL atlas was unable to label the dipole correctly due to its location in the WM, but the ICBM label was able to correctly identify it as WM since it includes WM parcellation; TD, Talairach Atlas; ICBM, International Consortium for Brain Mapping. Temporal Lob, temporal lobe.

Supplementary Table 1. List of all available atlases in MAV and their labeling systems. The MAV application uses the SPM MNI template to normalize the MRI and reverse-normalize it with available atlases, adapted from the Pickatlas application.

Atlas	Labeling System
AAL MNI v4/ AAL116	116 GM anatomical parcellations
Anterior Salience	7 anatomical-functional WM parcellations
Atlas116	116 GM anatomical parcellations
Atlas71	71 GM anatomical parcellations
Auditory	3 anatomical-functional WM parcellations
Basal Ganglia	5 anatomical-functional WM parcellations
CBF (Cerebral Blood Flow)	7 major cerebral arterial territories parcellations
Dorsal DMN (default mode network)	9 anatomical-functional WM parcellations
High Visual	2 anatomical-functional WM parcellations
ICBM (International Consortium for Brain Mapping)	56 GM + WM anatomical parcellations
ICBM WMPM (White Matter Parcellation Map)	50 WM anatomical parcellations
Language	7 anatomical-functional WM parcellations
LECN (left Executive Control Network)	6 anatomical-functional WM parcellations
Posterior Salience	12 anatomical-functional WM parcellations
Precuneus	4 anatomical-functional WM parcellations
Primary Visual	2 anatomical-functional WM parcellations
rECN (right Executive Control Network	6 anatomical-functional WM parcellations
Sensorimotor	6 anatomical-functional WM parcellations
TD (Talaraich Daemon) Brodmann	47 Broadmann areas + 28 subcortical anatomical parcellations
TD (Talaraich Daemon) label	55 GM + Ventricle anatomical parcellations
Talaraich Daemon (TD) lobe	12 anatomical structures: lobes + subcortical structures
Visuospatial	11 anatomical-functional WM parcellations

GM, gray matter; WM, white matter.
